# The *in vitro* activity of aztreonam-avibactam and cefiderocol against globally collected clinical metallo-β-lactamase- and/or serine-carbapenemase-positive Enterobacterales isolates and the utility of commonly used *in vitro* diagnostic kits

**DOI:** 10.1128/spectrum.03820-25

**Published:** 2026-03-31

**Authors:** Mark Estabrook, Christina Streit, Andrew Townsend, Gregory Stone, Shweta Kamat, Daniel Sahm

**Affiliations:** 1 IHMA 167022 , Schaumburg, Illinois, USA; 2 Pfizer Inc 357389 , Tadworth, United Kingdom; 3 Pfizer Inc 105623 , Groton, Connecticut, USA; 4 Pfizer PIPL 732625 , Mumbai, India; Universidad de Buenos Aires, Buenos Aires, Argentina

**Keywords:** aztreonam-avibactam, cefiderocol, Carba 5, Carba-R, IVD, carbapenemase, metallo-beta-lactamase, carbapenem-resistant Enterobacterales

## Abstract

The NG-test CARBA 5 and Xpert Carba-R IVD kits were tested against 269 clinical Enterobacterales isolates selected for their previously determined carbapenemase content from the ATLAS Global Surveillance program, primarily from 2020 to 2022. Antimicrobial susceptibility testing by broth microdilution was performed for aztreonam-avibactam, cefiderocol, and othe agents using EUCAST 2025 breakpoints for interpretation. The CARBA 5 and Carba-R IVD kits accurately identified all NDM (93), KPC (36), and OXA-48-like (43) carbapenemases. The CARBA 5 detected all IMP (42) and 45/47 VIM, and the Carba-R detected all VIM (47) and 21/42 IMP. Aztreonam-avibactam was active against carbapenemase-producing isolates (percent susceptible [carbapenemases]): 90.7% (NDM), 92.9% (NDM+OXA-48-like), 100% (IMP, VIM, and KPC), and 88.9% (OXA-48-like). Cefiderocol susceptibility was as follows: 38.7% (NDM), 50.0% (NDM+OXA-48-like), 77.5% (IMP), 82.5% (VIM), 75.9% (KPC), and 100% (OXA-48-like). The most common carbapenemases in Enterobacterales include NDM, KPC, and OXA-48-like enzymes, all of which were accurately identified by the CARBA 5 and Carba-R kits. Aztreonam-avibactam demonstrated potent *in vitro* activity against isolates determined to be positive for carbapenemases by these kits, offering a potential therapeutic option when one of these isolates is encountered in the clinic.

**IMPORTANCE:**

*In vitro* diagnostic (IVD) kits exist that detect carbapenemases in clinical isolates of Enterobacterales. Their utility is hampered by the limited options to reliably treat infections caused by Enterobacterales that produce metallo-β-lactamases. Here, we test the utility of IVD kits for detecting carbapenemases in a recent collection of clinical Enterobacterales isolates and determine the *in vitro* activity of aztreonam-avibactam, cefiderocol, and other comparator agents against isolates that were positive for carbapenemases.

## INTRODUCTION

Enterobacterales cause a significant proportion of urinary tract infections (UTIs), hospital/ventilator-acquired bacterial pneumonia (HABP/VABP), intra-abdominal infections (IAIs), and other types of infections (1, 2). Antimicrobial resistance among Enterobacterales has been increasing globally (3, 4) and threatens the effective treatment of the deadly infections caused by these organisms. In recent years, the rapid global dissemination of carbapenem-resistant Enterobacterales (CRE) and particularly those that produce metallo-β-lactamases (MBLs) has been a primary concern (3). MBLs hydrolyze all classes of clinically available β-lactam antibiotics except for monobactams such as aztreonam (5).

Several *in vitro* diagnostic (IVD) kits are available that detect MBLs and serine-carbapenemases from bacterial isolates and provide the opportunity to switch to more effective therapies before gold standard culture-based susceptibility testing results can be obtained (6). Among those commonly used in clinical practice are the Xpert Carba-R (Cepheid) and the NG-Test CARBA 5 (Hardy Diagnostics), both of which can detect the most common MBLs in Enterobacterales isolates: NDM, IMP, and VIM, as well as the common families of serine-carbapenemases, KPC and OXA-48-like (6).

Beyond the detection of carbapenemases in an isolate of Enterobacterales lies the challenge of selecting an effective therapy for treatment before antimicrobial susceptibility data can be obtained. Aztreonam-avibactam is a combination of the monobactam aztreonam, which is stable to hydrolysis by MBLs, with the diazabicyclooctane β-lactamase inhibitor avibactam, which has inhibitory activity against Class A, C, and some Class D β-lactamases that are frequently co-produced with MBLs in clinical isolates of Enterobacterales (7). Aztreonam-avibactam has been authorized for use by the European Medicines Agency (EMA) for the treatment of adults with complicated IAIs, HABP/VABP, complicated UTIs, and infections caused by gram-negative bacteria with limited treatment options (8). Aztreonam-avibactam has also been approved by the United States Food and Drug Administration (U.S. FDA) for the treatment of adults with complicated IAI with limited or no treatment options (9). Global surveillance data have shown that aztreonam-avibactam is potent against MBL-producing Enterobacterales isolates *in vitro* (10).

Cefiderocol is another therapeutic option that has been authorized for use by the EMA for infections caused by aerobic gram-negative bacteria in adults with limited treatment options (11). Cefiderocol is also approved by the U.S. FDA for the treatment of complicated UTI, including pyelonephritis, and HABP/VABP caused by susceptible gram-negative organisms in adult patients with limited treatment options (12). Cefiderocol is a first-in-class siderophore cephalosporin that enters the bacterial cell through active transport mechanisms associated with iron acquisition (13). While cefiderocol can be hydrolyzed by MBLs such as NDM and other β-lactamases, it has shown *in vitro* activity against some organisms with these mechanisms in part due to its unique mode of entry (14, 15).

Here, we selected isolates of known carbapenemase gene carriage from the ATLAS Global Surveillance Program to test the sensitivity of the CARBA 5 and Carba-R IVD kits and determined the *in vitro* activity of aztreonam-avibactam, cefiderocol, and other comparator agents against these isolates.

## MATERIALS AND METHODS

### Enterobacterales isolates

**Fig 1:**
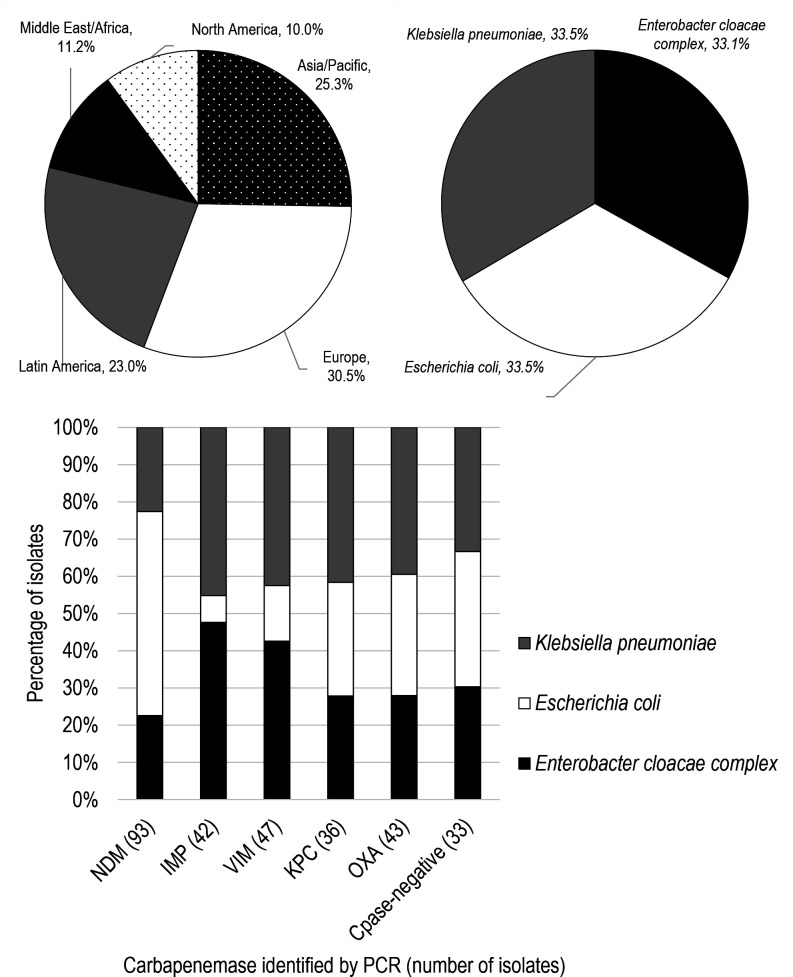
Demographics of isolates in this study. OXA: OXA-48-like carbapenemases; Cpase: carbapenemase.

An even distribution of *Escherichia coli* (*n* = 90)*, Klebsiella pneumoniae* (*n* = 90), and *Enterobacter cloacae* complex (*n* = 89) isolates was selected from the ATLAS Global Surveillance program based on previously determined carbapenemase gene carriage, which was determined using a multiplex PCR and subsequent Sanger sequencing (16) (Fig. 1). Isolates were positive for *bla_NDM_* (*n* = 93), *bla_IMP_* (*n* = 42), *bla_VIM_* (*n* = 47), *bla_KPC_* (*n* = 36), and *bla_OXA-48-like_* (*n* = 43) or had no detected carbapenemase gene (*n* = 33) (Fig. 1). Isolates selected carried a single carbapenemase gene (*n* = 211) or multiple carbapenemase genes (*n* = 25). Fourteen isolates carried *bla_NDM_+bla_OXA-48-like_* and 11 carried other combinations of carbapenemase genes (Table S1). Isolates were collected between 2013 and 2022, with 242/269 collected from 2020 to 2022. Older isolates were included to provide adequate numbers of those carrying carbapenemase genes that are less common in Enterobacterales (IMP and VIM).

### Detection of β-lactamases and β-lactamase-encoding genes

The Cepheid Xpert Carba-R (Carba-R) and Hardy Diagnostics NG-Test CARBA 5 (CARBA 5) kits were run using a 0.5 McFarland suspension of bacteria in saline. Bacteria were subcultured on tryptic soy agar plates with 5% sheep’s blood (Remel) prior to suspension. Confirmatory multiplex PCR for carbapenemase genes was run on DNA isolated from the same analyte tested using the IVDs as described previously (16). For analysis, where antimicrobial susceptibility is described, isolates co-carrying multiple carbapenemase genes are shown in categories separate from those that carry a single carbapenemase gene. Where the carbapenemase-positivity by IVD kit is described, isolates positive for multiple carbapenemases are counted as positive for each relevant carbapenemase without addressing co-carriage.

### Antimicrobial-susceptibility testing (AST)

AST was performed using the broth microdilution method described by the (CLSI 2024) guidelines (17). Interpretations of MIC data are provided according to the EUCAST 2025 breakpoints (18). Cefiderocol was tested using ion-depleted cation-adjusted Mueller-Hinton broth (CAMHB), and all other agents were tested with CAMHB. Avibactam was tested at a fixed concentration of 4 µg/mL.

## RESULTS

### Detection of carbapenemases by IVD kits

The CARBA 5 and Carba-R kits never demonstrated a false-positive result using the PCR assay as a gold standard (Table 1). Both kits also positively identified every NDM, KPC, and OXA-48-like carbapenemase that was identified by PCR.

**TABLE 1:** Agreement of IVD kits with PCR, by β-lactamase variant^*a*^

Carbapenemase variant	n	CARBA 5		Carba-R	
		*n* pos.	% agreement	*n* pos.	% agreement
Any NDM	93	93	100	93	100
NDM-5	46	46	100	46	100
NDM-1	40	40	100	40	100
NDM-7	5	5	100	5	100
NDM-4	2	2	100	2	100
No NDM	176	0	100	0	100
Any IMP	42	42	100	21	50.0
IMP-8	18	18	100	0	0.0
IMP-4	11	11	100	11	100
IMP-26	6	6	100	6	100
IMP-1	2	2	100	2	100
IMP-14	2	2	100	0	0.0
IMP-10	1	1	100	1	100
IMP-13	1	1	100	0	0.0
IMP-59	1	1	100	1	100
No IMP	227	0	100	0	100
Any VIM	47	45	95.7	47	100
VIM-1	37	37	100	37	100
VIM-4	4	4	100	4	100
VIM-23	3	2	66.7	3	100
VIM-24	2	1	50.0	2	100
VIM-2	1	1	100	1	100
No VIM	222	0	100	0	100
Any KPC	36	36	100	36	100
KPC-2	25	25	100	25	100
KPC-3	10	10	100	10	100
KPC-2+33	1	1	100	1	100
No KPC	233	0	100	0	100
Any OXA-48-like	43	43	100	43	100
OXA-48	15	15	100	15	100
OXA-181	13	13	100	13	100
OXA-232	8	8	100	8	100
OXA-244	3	3	100	3	100
OXA-370	3	3	100	3	100
OXA-1205	1	1	100	1	100
No OXA-48-like	226	0	100	0	100

^*a*^ PCR: PCR+Sanger sequencing as previously described (16); CARBA 5, Hardy NG-Test CARBA 5; Carba-R, Cepheid Xpert Carba-R; POS, positive, NEG, negative.

The CARBA 5 kit failed to detect variants of VIM in two of the 47 isolates that were positive by PCR and by the Carba-R kit. The variants of VIM missed by the CARBA 5 kit were VIM-23 and VIM-24, both of which were detected in other isolates by this kit.

The Carba-R kit failed to detect variants of IMP in 21 of 42 isolates that were positive by PCR and the CARBA 5 kit. The variants missed were IMP-8 (*n* = 18), IMP-14 (*n* = 2), and IMP-13 (*n* = 1). In contrast with the CARBA 5 kit, there was no case where the same variant of a β-lactamase was sometimes detected and sometimes not detected in different isolates by the Carba-R kit.

### The *in vitro* activity of aztreonam-avibactam, cefiderocol, and comparator agents against isolates positive for carbapenemase genes

The percentage of isolates susceptible to aztreonam-avibactam among those genotyped by PCR were (percent [carbapenemase]): 90.7% (NDM), 92.9% (NDM+OXA-48-like), 100% (IMP), 100% (VIM), 100% (KPC), 88.9% (OXA-48-like), 90.9% (Other/multiple), and 100% (no carbapenemase) (Table 2). Aztreonam-avibactam was active against more isolates *in vitro* than cefiderocol, with the exception of isolates producing OXA-48-like carbapenemases (and no NDM), of which all were susceptible to cefiderocol, and 24/27 were susceptible to aztreonam-avibactam. Other comparators demonstrated susceptibility rates against any subset of carbapenemase-positive isolates of 0.0%–60.0% (meropenem), 42.9%–90.0% (amikacin), or 72.7%–100% (colistin), depending on the carbapenemase genotype.

**TABLE 2:** *In vitro* activities of aztreonam-avibactam and comparator agents against Enterobacterales isolates, by genotype determined by PCR+Sanger sequencing^*a*^^,^^*e*^

Carbapenemase genotype (PCR)^*b*^	*n*	Agent [MIC_90_ (µg/mL), percent susceptible^*c*^]									
		ATM-AVI		FDC		MEM		AMK		COL	
		MIC_90_	%S	MIC_90_	%S	MIC_90_	%S	MIC_90_	%S	MIC_90_	%S
NDM	75	4	**90.7**	32	38.7	64	1.3	>16	66.7	0.5	**92.0**
NDM+OXA-48-like	14	1	**92.9**	16	50.0	>64	7.1	>16	42.9	0.5	**100**
IMP	40	0.5	**100**	4	77.5	16	60.0	>16	82.5	0.5	**97.5**
VIM	40	0.5	**100**	4	82.5	32	32.5	8	**90.0**	0.5	**92.5**
KPC	29	0.5	**100**	4	75.9	>64	17.2	>16	72.4	>8	79.3
OXA-48-like	27	8	**88.9**	2	**100**	32	51.9	>16	77.8	0.5	**96.3**
Other/multiple^*d*^	11	1	**90.9**	8	54.5	>64	0.0	>16	54.5	>8	72.7
None detected	33	2	**100**	2	**90.9**	2	**93.9**	4	**97.0**	0.5	**90.9**

^*a*^ ATM-AVI, aztreonam-avibactam; FDC, cefiderocol; MEM, meropenem; AMK, amikacin; COL, colistin.

^*b*^ A multiplex PCR followed by full-length PCR amplification and Sanger sequencing (16), followed by confirmation with Sanger sequencing.

^*c*^ Percent susceptible using EUCAST 2025 breakpoints.

^*d*^ Other/multiple includes isolates that co-carried VIM and KPC (4), VIM and NDM (2), VIM and OXA-48-like (1), IMP and KPC (1), IMP and NDM (1), KPC and OXA-48-like (1), and NDM and KPC (1).

^*e*^ Values in bold are at least 90%.

Of 177 isolates positive for any MBL by the CARBA 5 kit, 95.5% were susceptible to aztreonam-avibactam, and 58.8% were susceptible to cefiderocol (Table 3). Of 159 isolates positive for any MBL by the Carba-R kit, 95.0% were susceptible to aztreonam-avibactam, and 57.2% were susceptible to cefiderocol. The carbapenemase genotype associated with the greatest difference in percent susceptible to aztreonam-avibactam and cefiderocol was NDM, of which 91% were susceptible to aztreonam-avibactam and 38%–39% were susceptible to cefiderocol.

**TABLE 3:** *In vitro* activities of aztreonam-avibactam and comparator agents against isolates with carbapenemases identified by the CARBA 5 and Carba-R IVD kits^*a*^

β-lactamases	No. isolates (CARBA 5 | Carba-R)	Percent susceptible to agent^*b*^ (IVD kit used to identify β-lactamase genotype)									
		ATM-AVI		FDC		MEM		AMK		COL	
		CARBA 5	Carba-R	CARBA 5	Carba-R	CARBA 5	Carba-R	CARBA 5	Carba-R	CARBA 5	Carba-R
Any MBL	177 | 159	**95.5**	**95.0**	58.8	57.2	21.5	14.5	73.4	72.3	**93.8**	**91.8**
NDM	75 | 76	**90.7**	**90.8**	38.7	38.2	1.3	1.3	66.7	67.1	**92.0**	**92.1**
NDM+OXA-48-like	14 | 14	**92.9**	**92.9**	50.0	50.0	7.1	7.1	42.9	42.9	**100**	**100**
IMP	40 | 20	**100**	**100**	77.5	80.0	60.0	40.0	82.5	85.0	**97.5**	**95.0**
VIM	39 | 40	**100**	**100**	77.5	80.0	30.8	32.5	89.7	**90.0**	**94.9**	**92.5**
KPC	30 | 29	**100**	**100**	76.7	75.9	16.7	17.2	70.0	72.4	76.7	79.3
OXA-48-like	27 | 27	88.9	88.9	**100**	**100**	51.9	51.9	77.8	77.8	**96.3**	**96.3**
Multiple/Other	10 | 10	**90.0**	**90.0**	50.0	60.0	0.0	0.0	60.0	50.0	80.0	70.0

^*a*^ ATM-AVI, aztreonam-avibactam; FDC, cefiderocol; MEM, meropenem; AMK, amikacin; COL, colistin. Values in bold are ≥90.0%.

^*b*^ Percent susceptible using EUCAST 2025 breakpoints.

### Characteristics of aztreonam-avibactam-resistant isolates

Of the 12 isolates that were resistant to aztreonam-avibactam, 11 were *E. coli* (92%), 9 were collected in India (75%), all were collected in 2022 (100%), and 10 carried a gene encoding non-intrinsic AmpC (83.3%) (Table S2). Of the isolates that carried AmpC, CMY was the most common (*n* = 9), and variants of CMY were CMY-42 (*n* = 4), CMY-145 (*n* = 2), CMY-4 (*n* = 1), or a novel variant (*n* = 2). These isolates were not queried for insertion sequences in PBP3, which have previously been shown to contribute to aztreonam-avibactam resistance (19, 20).

## DISCUSSION

Against this collection of global clinical isolates of taxa within Enterobacterales that most frequently produce carbapenemases (3), the CARBA 5 and Carba-R kits demonstrated complete agreement with each other and the PCR assay employed for the detection of the most common carbapenemases in Enterobacterales: NDM, KPC, and OXA-48-like (3). The results for less common carbapenemases, VIM and IMP, demonstrated some discordance for the CARBA 5 and Carba-R, respectively.

Of note, this study was designed to test the detection of carbapenemases by IVD kits and show the *in vitro* activity of aztreonam-avibactam, cefiderocol, and comparator agents. The proportion of species with carbapenemases and the families of carbapenemases included in the study are not intended to match the prevalence of these organisms among CRE or the types of carbapenemases they produce in clinical settings.

Issues with the CARBA 5 assay detecting VIM have been reported previously and can be variable depending on the media used to subculture the bacterial isolate (21). In the previous study, VIM was not detected in 1/11 (9.1%) of VIM-positive isolates, compared to 2/47 (4.3%) of VIM-positive isolates in the present study. The isolates that were VIM-positive by PCR and Carba-R, for which no VIM was detected by the CARBA 5 kit, carried genes encoding VIM-23 and VIM-24. Other isolates producing these variants of VIM were positive by the CARBA 5 kit, indicating the failure to detect these carbapenemases is not specific to the variant, possibly implicating gene expression levels or some other phenomenon in the variability of detection for this enzyme.

The Xpert Carba-R product insert states that select variants of IMP (encoded by *bla_IMP-7 -13_ or _-14_*) are not detected by this IVD kit (22). The genes that were not detected in this study were *bla_IMP-8, -13,_* and *_-14_*. The ability of the kit to detect *bla_IMP-8_* is not discussed in the package insert, but other authors report failure to detect this variant with the Carba-R kit (23, 24).

The increasing incidence of isolates that co-carry NDM+OXA-48-like carbapenemases has been noted in other works (4). In this study, all 14 isolates that carried NDM+OXA-48-like carbapenemases were correctly identified by the CARBA 5 and Carba-R kits, 13/14 were susceptible to aztreonam-avibactam, and 7/14 were susceptible to cefiderocol.

Aztreonam-avibactam demonstrated potent *in vitro* activity against isolates that carried carbapenemases, including those that were positive using the CARBA 5 and Carba-R diagnostic kits. Cefiderocol activity was potent against those carrying OXA-48-like carbapenemases, but ≤59% of isolates carrying any MBL detected by the CARBA 5 kit or the Carba-R kit were susceptible to cefiderocol using EUCAST breakpoints. The activity of cefiderocol against NDM-producing organisms was lower, yet with ≤39% of isolates susceptible as compared to 91% of these NDM-producing isolates susceptible to aztreonam-avibactam. Utilization of IVD kits paired with knowledge of the *in vitro* activity of newly available agents is one of many important ways to inform selection of appropriate therapy and improve patient outcomes in a challenging landscape of antimicrobial resistance.

## Data Availability

The complete antimicrobial susceptibility testing data and results of IVD kits are included in the Supplemental tables. External sequence data consisted solely of the Bacterial Antimicrobial Resistance Reference Gene Database hosted by the National Center for Biotechnology Information (NCBI), bioproject PRJNA313047.

## SUPPLEMENTAL MATERIAL

Supplemental tables spectrum.03820-25-s0001.docx Tables S1 to S3.

ASM does not own the copyrights to Supplemental Material that may be linked to, or accessed through, an article. The authors have granted ASM a non-exclusive, world-wide license to publish the Supplemental Material files. Please contact the corresponding author directly for reuse.
