# Supplementary material for: The in vitro activity of aztreonam-avibactam and cefiderocol against globally collected clinical metallo-β-lactamase- and/or serine-carbapenemase-positive Enterobacterales isolates and the utility of commonly used in vitro diagnostic kits
Source: Microbiol Spectr. 2026 Mar 31;14(5):e03820-25. doi: 10.1128/spectrum.03820-25 (PMC13141907; doi:10.1128/spectrum.03820-25)
Supplement: Supplemental tables — Tables S1 to S3. [file spectrum.03820-25-s0001.docx]

1. **Supplementary Tables**

## Supplementary Table 1. Carbapenemase genes detected by PCR in isolates that carry multiple carbapenemases

| **Organism/carbapenemase genotype** | **n** |
| --- | --- |
| *Enterobacter cloacae* complex | 2 |
| VIM+NDM | 1 |
| VIM+OXA-48-like | 1 |
| *Escherichia coli* | 3 |
| IMP+NDM | 1 |
| KPC+OXA-48-like | 1 |
| NDM+KPC | 1 |
| *Klebsiella pneumoniae* | 6 |
| IMP+KPC | 1 |
| VIM+KPC | 4 |
| VIM+NDM | 1 |
| Grand Total | 11 |

## Supplementary Table 2. Demographic and β-lactamase summary (including non-carbapenemase) for aztreonam-avibactam-resistant isolates.

| **Isolate** | **Year** | **Country** | **Organism** | **CARBA 5 result** | **Carba-R result** | **PCR β-lactamase summary** | **Agent [MIC (µg/mL)]** | | | | |  |
| --- | --- | --- | --- | --- | --- | --- | --- | --- | --- | --- | --- | --- |
|  |  |  |  |  |  |  | **ATM-AVI** | **FDC** | **MEM** | **AMK** | **COL** | |
| 22-003858-01 | 2022 | India | *E. coli* | NDM | NDM | TEM-OSBL; CMY-New variant; NDM-5 | >32 | 4 | 64 | 2 | ≤0.25 | |
| 22-031312-01 | 2022 | India | *E. coli* | NDM | NDM | CMY-New variant; NDM-5 | >32 | 8 | 64 | 2 | ≤0.25 | |
| 22-015013-01 | 2022 | India | *E. coli* | KPC+OXA-48-like | KPC+OXA-48-like | VEB-New variant; CMY-4; KPC-2; OXA-181 | 16 | >64 | 4 | >16 | ≤0.25 | |
| 22-003994-01 | 2022 | India | *E. coli* | OXA48 | OXA48 | CTX-M-15; CMY-145; OXA-1205 | 16 | 1 | 0.12 | 4 | ≤0.25 | |
| 21-000961-01 | 2022 | India | *E. coli* | NDM | NDM | CTX-M-15; DHA-1; NDM-5 | 8 | 4 | 32 | 4 | ≤0.25 | |
| 22-012880-01 | 2022 | India | *E. coli* | NDM | NDM | NDM-5 | 8 | 4 | 64 | 4 | ≤0.25 | |
| 22-017050-01 | 2022 | Mexico | *E. coli* | NDM | NDM | CMY-42; NDM-5 | 8 | 8 | 64 | 1 | ≤0.25 | |
| 22-031313-01 | 2022 | India | *E. coli* | NDM | NDM | TEM-OSBL; CMY-145; NDM-5 | 8 | >64 | >64 | 2 | ≤0.25 | |
| 22-031674-01 | 2022 | India | *E. coli* | NDM | NDM | TEM-OSBL; CTX-M-15; CMY-42; NDM-5 | 8 | 4 | 32 | >16 | ≤0.25 | |
| 21-003645-01 | 2022 | India | *E. coli* | NDM+OXA48 | NDM+OXA48 | TEM-OSBL; CTX-M-15; CMY-42; OXA-181; NDM-5 | 8 | 16 | 64 | >16 | 0.5 | |
| 21-002808-01 | 2022 | Thailand | *K. pneumoniae* | OXA48 | OXA48 | TEM-OSBL; SHV-OSBL; CTX-M-15; OXA-232 | 8 | 2 | 32 | >16 | >8 | |
| 22-021597-01 | 2022 | Turkey | *E. coli* | OXA48 | OXA48 | TEM-OSBL; CMY-42; OXA-181 | 8 | 1 | 32 | 4 | ≤0.25 | |

## **Supplementary Table 3. PCR, Carba5, and CarbaR results**

| **IHMANumber** | **Carba5 Result** | **CarbaR result** | **Reference PCR** |
| --- | --- | --- | --- |
| 24-138113-01 | IMP | IMP | IMP |
| 24-138114-01 | IMP | IMP | IMP |
| 24-138116-01 | IMP | IMP | IMP |
| 24-138115-01 | IMP | Negative | IMP |
| 24-138118-01 | IMP | IMP | IMP |
| 24-138119-01 | IMP | IMP | IMP |
| 24-138120-01 | IMP | IMP | IMP |
| 24-138121-01 | IMP | IMP | IMP |
| 24-138124-01 | IMP | Negative | IMP |
| 24-138125-01 | IMP | Negative | IMP |
| 24-011457-01 | IMP | IMP | IMP |
| 24-011461-01 | IMP | IMP | IMP |
| 24-011463-01 | IMP | IMP | IMP |
| 24-138126-01 | IMP | IMP | IMP |
| 24-138127-01 | IMP | Negative | IMP |
| 24-138129-01 | IMP | Negative | IMP |
| 24-011726-01 | IMP | IMP | IMP |
| 24-138185-01 | IMP | IMP | IMP |
| 24-138177-01 | IMP | Negative | IMP |
| 24-138181-01 | IMP | Negative | IMP |
| 24-138182-01 | IMP | Negative | IMP |
| 24-011657-01 | IMP | IMP | IMP |
| 24-011661-01 | IMP | IMP | IMP |
| 24-011656-01 | IMP | Negative | IMP |
| 24-011659-01 | IMP | Negative | IMP |
| 24-011660-01 | IMP | Negative | IMP |
| 24-011665-01 | IMP | Negative | IMP |
| 21-036597-01 | IMP | Negative | IMP |
| 21-036655-01 | IMP | Negative | IMP |
| 21-036681-01 | IMP | Negative | IMP |
| 22-026928-01 | IMP | Negative | IMP |
| 22-033140-01 | IMP | Negative | IMP |
| 22-033210-01 | IMP | Negative | IMP |
| 22-063987-01 | IMP | Negative | IMP |
| 22-063999-01 | IMP | Negative | IMP |
| 24-138108-01 | IMP | IMP | IMP |
| 24-138109-01 | IMP | IMP | IMP |
| 24-138110-01 | IMP | IMP | IMP |
| 24-138111-01 | IMP | IMP | IMP |
| 24-138107-01 | IMP | IMP | IMP |
| 24-138186-01 | IMP+KPC | IMP+KPC | IMP+KPC |
| 24-011662-01 | IMP+NDM | NDM | IMP+NDM |
| 24-138190-01 | KPC | KPC | KPC |
| 24-138191-01 | KPC | KPC | KPC |
| 24-138192-01 | KPC | KPC | KPC |
| 21-006683-01 | KPC | KPC | KPC |
| 21-016458-01 | KPC | KPC | KPC |
| 21-016469-01 | KPC | KPC | KPC |
| 21-034147-01 | KPC | KPC | KPC |
| 21-034271-01 | KPC | KPC | KPC |
| 21-044328-01 | KPC | KPC | KPC |
| 21-044945-01 | KPC | KPC | KPC |
| 22-019431-01 | KPC | KPC | KPC |
| 22-020195-01 | KPC | KPC | KPC |
| 22-027497-01 | KPC | KPC | KPC |
| 22-027543-01 | KPC | KPC | KPC |
| 22-031709-01 | KPC | KPC | KPC |
| 22-032412-01 | KPC | KPC | KPC |
| 22-042814-01 | KPC | KPC | KPC |
| 22-045588-01 | KPC | KPC | KPC |
| 22-049914-01 | KPC | KPC | KPC |
| 22-059434-01 | KPC | KPC | KPC |
| 22-059511-01 | KPC | KPC | KPC |
| 22-059723-01 | KPC | KPC | KPC |
| 22-060022-01 | KPC | KPC | KPC |
| 22-060072-01 | KPC | KPC | KPC |
| 22-064336-01 | KPC | KPC | KPC |
| 22-064638-01 | KPC | KPC | KPC |
| 22-064738-01 | KPC | KPC | KPC |
| 22-065838-01 | KPC | KPC | KPC |
| 24-142380-01 | KPC | KPC | KPC |
| 22-015013-01 | KPC+OXA-48-like | KPC+OXA-48-like | KPC+OXA-48-like |
| 24-011571-01 | NDM | NDM | NDM |
| 24-011572-01 | NDM | NDM | NDM |
| 24-011575-01 | NDM | NDM | NDM |
| 24-011610-01 | NDM | NDM | NDM |
| 24-112255-01 | NDM | NDM | NDM |
| 21-000953-01 | NDM | NDM | NDM |
| 21-000961-01 | NDM | NDM | NDM |
| 21-001472-01 | NDM | NDM | NDM |
| 21-001634-01 | NDM | NDM | NDM |
| 21-002852-01 | NDM | NDM | NDM |
| 21-003656-01 | NDM | NDM | NDM |
| 21-005535-01 | NDM | NDM | NDM |
| 21-007284-01 | NDM | NDM | NDM |
| 21-007363-01 | NDM | NDM | NDM |
| 21-013851-01 | NDM | NDM | NDM |
| 21-014675-01 | NDM | NDM | NDM |
| 21-015422-01 | NDM | NDM | NDM |
| 21-015517-01 | NDM | NDM | NDM |
| 21-016544-01 | NDM | NDM | NDM |
| 21-017194-01 | NDM | NDM | NDM |
| 21-018016-01 | NDM | NDM | NDM |
| 21-032510-01 | NDM | NDM | NDM |
| 21-038042-01 | NDM | NDM | NDM |
| 21-038111-01 | NDM | NDM | NDM |
| 21-042158-01 | NDM | NDM | NDM |
| 21-051301-01 | NDM | NDM | NDM |
| 22-003858-01 | NDM | NDM | NDM |
| 22-009609-01 | NDM | NDM | NDM |
| 22-009658-01 | NDM | NDM | NDM |
| 22-010103-01 | NDM | NDM | NDM |
| 22-010267-01 | NDM | NDM | NDM |
| 22-010377-01 | NDM | NDM | NDM |
| 22-011407-01 | NDM | NDM | NDM |
| 22-012880-01 | NDM | NDM | NDM |
| 22-013194-01 | NDM | NDM | NDM |
| 22-015701-01 | NDM | NDM | NDM |
| 22-017050-01 | NDM | NDM | NDM |
| 22-018987-01 | NDM | NDM | NDM |
| 22-019113-01 | NDM | NDM | NDM |
| 22-019425-01 | NDM | NDM | NDM |
| 22-019459-01 | NDM | NDM | NDM |
| 22-020544-01 | NDM | NDM | NDM |
| 22-020561-01 | NDM | NDM | NDM |
| 22-022186-01 | NDM | NDM | NDM |
| 22-022205-01 | NDM | NDM | NDM |
| 22-022441-01 | NDM | NDM | NDM |
| 22-024108-01 | NDM | NDM | NDM |
| 22-025210-01 | NDM | NDM | NDM |
| 22-027729-01 | NDM | NDM | NDM |
| 22-027750-01 | NDM | NDM | NDM |
| 22-027855-01 | NDM | NDM | NDM |
| 22-028055-01 | NDM | NDM | NDM |
| 22-029754-01 | NDM | NDM | NDM |
| 22-031312-01 | NDM | NDM | NDM |
| 22-031313-01 | NDM | NDM | NDM |
| 22-031674-01 | NDM | NDM | NDM |
| 22-033430-01 | NDM | NDM | NDM |
| 22-037259-01 | NDM | NDM | NDM |
| 22-041975-01 | NDM | NDM | NDM |
| 22-059178-01 | NDM | NDM | NDM |
| 22-059420-01 | NDM | NDM | NDM |
| 22-060102-01 | NDM | NDM | NDM |
| 22-062893-01 | NDM | NDM | NDM |
| 22-064335-01 | NDM | NDM | NDM |
| 22-064340-01 | NDM | NDM | NDM |
| 22-064341-01 | NDM | NDM | NDM |
| 22-065120-01 | NDM | NDM | NDM |
| 22-065727-01 | NDM | NDM | NDM |
| 22-065995-01 | NDM | NDM | NDM |
| 22-076357-01 | NDM | NDM | NDM |
| 22-081244-01 | NDM | NDM | NDM |
| 22-084982-01 | NDM | NDM | NDM |
| 22-012887-01 | NDM | NDM | NDM |
| 22-017002-01 | NDM | NDM | NDM |
| 22-064345-01 | NDM | NDM | NDM |
| 22-037810-01 | NDM+KPC | NDM+KPC | NDM+KPC |
| 24-011554-01 | NDM+OXA48 | NDM+OXA48 | NDM+OXA-48-like |
| 24-037390-01 | NDM+OXA48 | NDM+OXA48 | NDM+OXA-48-like |
| 21-003645-01 | NDM+OXA48 | NDM+OXA48 | NDM+OXA-48-like |
| 21-043418-01 | NDM+OXA48 | NDM+OXA48 | NDM+OXA-48-like |
| 22-014394-01 | NDM+OXA48 | NDM+OXA48 | NDM+OXA-48-like |
| 22-015195-01 | NDM+OXA48 | NDM+OXA48 | NDM+OXA-48-like |
| 22-029567-01 | NDM+OXA48 | NDM+OXA48 | NDM+OXA-48-like |
| 22-031656-01 | NDM+OXA48 | NDM+OXA48 | NDM+OXA-48-like |
| 22-031680-01 | NDM+OXA48 | NDM+OXA48 | NDM+OXA-48-like |
| 22-031687-01 | NDM+OXA48 | NDM+OXA48 | NDM+OXA-48-like |
| 22-064509-01 | NDM+OXA48 | NDM+OXA48 | NDM+OXA-48-like |
| 22-065843-01 | NDM+OXA48 | NDM+OXA48 | NDM+OXA-48-like |
| 22-066336-01 | NDM+OXA48 | NDM+OXA48 | NDM+OXA-48-like |
| 22-076358-01 | NDM+OXA48 | NDM+OXA48 | NDM+OXA-48-like |
| 24-138193-01 | Negative | Negative | Negative |
| 24-011664-01 | Negative | Negative | Negative |
| 21-000921-01 | Negative | Negative | Negative |
| 21-001479-01 | Negative | Negative | Negative |
| 21-002944-01 | Negative | Negative | Negative |
| 21-014805-01 | Negative | Negative | Negative |
| 21-015358-01 | Negative | Negative | Negative |
| 21-033019-01 | Negative | Negative | Negative |
| 21-036163-01 | Negative | Negative | Negative |
| 21-042123-01 | Negative | Negative | Negative |
| 21-043438-01 | Negative | Negative | Negative |
| 21-043708-01 | Negative | Negative | Negative |
| 22-001213-01 | Negative | Negative | Negative |
| 22-007797-01 | Negative | Negative | Negative |
| 22-008965-01 | Negative | Negative | Negative |
| 22-011754-01 | Negative | Negative | Negative |
| 22-015080-01 | Negative | Negative | Negative |
| 22-017792-01 | Negative | Negative | Negative |
| 22-018989-01 | Negative | Negative | Negative |
| 22-019446-01 | Negative | Negative | Negative |
| 22-025265-01 | Negative | Negative | Negative |
| 22-029637-01 | Negative | Negative | Negative |
| 22-042212-01 | Negative | Negative | Negative |
| 22-042705-01 | Negative | Negative | Negative |
| 22-042839-01 | Negative | Negative | Negative |
| 22-043204-01 | Negative | Negative | Negative |
| 22-062037-01 | Negative | Negative | Negative |
| 22-063739-01 | Negative | Negative | Negative |
| 22-064031-01 | Negative | Negative | Negative |
| 22-064053-01 | Negative | Negative | Negative |
| 22-064399-01 | Negative | Negative | Negative |
| 22-065184-01 | Negative | Negative | Negative |
| 22-065401-01 | Negative | Negative | Negative |
| 24-138189-01 | OXA48 | OXA48 | OXA-48-like |
| 21-002807-01 | OXA48 | OXA48 | OXA-48-like |
| 21-002808-01 | OXA48 | OXA48 | OXA-48-like |
| 21-002851-01 | OXA48 | OXA48 | OXA-48-like |
| 21-015519-01 | OXA48 | OXA48 | OXA-48-like |
| 21-023317-01 | OXA48 | OXA48 | OXA-48-like |
| 21-036172-01 | OXA48 | OXA48 | OXA-48-like |
| 21-042480-01 | OXA48 | OXA48 | OXA-48-like |
| 21-042491-01 | OXA48 | OXA48 | OXA-48-like |
| 21-043778-01 | OXA48 | OXA48 | OXA-48-like |
| 22-002660-01 | OXA48 | OXA48 | OXA-48-like |
| 22-003994-01 | OXA48 | OXA48 | OXA-48-like |
| 22-005469-01 | OXA48 | OXA48 | OXA-48-like |
| 22-021597-01 | OXA48 | OXA48 | OXA-48-like |
| 22-031320-01 | OXA48 | OXA48 | OXA-48-like |
| 22-031470-01 | OXA48 | OXA48 | OXA-48-like |
| 22-032369-01 | OXA48 | OXA48 | OXA-48-like |
| 22-045348-01 | OXA48 | OXA48 | OXA-48-like |
| 22-046687-01 | OXA48 | OXA48 | OXA-48-like |
| 22-046695-01 | OXA48 | OXA48 | OXA-48-like |
| 22-047352-01 | OXA48 | OXA48 | OXA-48-like |
| 22-047485-01 | OXA48 | OXA48 | OXA-48-like |
| 22-064398-01 | OXA48 | OXA48 | OXA-48-like |
| 22-070726-01 | OXA48 | OXA48 | OXA-48-like |
| 22-070727-01 | OXA48 | OXA48 | OXA-48-like |
| 22-081301-01 | OXA48 | OXA48 | OXA-48-like |
| 22-083116-01 | OXA48 | OXA48 | OXA-48-like |
| 24-138112-01 | VIM | VIM | VIM |
| 24-138117-01 | VIM | VIM | VIM |
| 24-011386-01 | VIM | VIM | VIM |
| 24-138128-01 | VIM | VIM | VIM |
| 24-138130-01 | VIM | VIM | VIM |
| 24-138131-01 | VIM | VIM | VIM |
| 24-138184-01 | Negative | VIM | VIM |
| 24-138176-01 | VIM | VIM | VIM |
| 24-138178-01 | VIM | VIM | VIM |
| 24-138179-01 | VIM | VIM | VIM |
| 24-138180-01 | VIM | VIM | VIM |
| 24-138183-01 | VIM | VIM | VIM |
| 24-138187-01 | VIM | VIM | VIM |
| 24-011677-01 | VIM | VIM | VIM |
| 24-132736-01 | VIM | VIM | VIM |
| 24-132744-01 | VIM | VIM | VIM |
| 24-138188-01 | VIM | VIM | VIM |
| 21-013613-01 | VIM | VIM | VIM |
| 21-016542-01 | VIM | VIM | VIM |
| 21-023271-01 | VIM | VIM | VIM |
| 21-038931-01 | VIM | VIM | VIM |
| 22-014765-01 | VIM | VIM | VIM |
| 22-014775-01 | VIM | VIM | VIM |
| 22-015189-01 | VIM | VIM | VIM |
| 22-033395-01 | VIM | VIM | VIM |
| 22-044892-01 | VIM | VIM | VIM |
| 22-044896-01 | VIM | VIM | VIM |
| 22-045603-01 | VIM | VIM | VIM |
| 22-045689-01 | VIM | VIM | VIM |
| 22-065198-01 | VIM | VIM | VIM |
| 22-067242-01 | VIM | VIM | VIM |
| 22-083325-01 | VIM | VIM | VIM |
| 22-083327-01 | VIM | VIM | VIM |
| 22-083328-01 | VIM | VIM | VIM |
| 22-083329-01 | VIM | VIM | VIM |
| 22-083330-01 | VIM | VIM | VIM |
| 22-083421-01 | VIM | VIM | VIM |
| 22-083424-01 | VIM | VIM | VIM |
| 22-083426-01 | VIM | VIM | VIM |
| 24-142381-01 | VIM | VIM | VIM |
| 24-011533-01 | KPC | VIM+KPC | VIM+KPC |
| 22-013158-01 | VIM+KPC | VIM+KPC | VIM+KPC |
| 22-013188-01 | VIM+KPC | VIM+KPC | VIM+KPC |
| 22-065888-01 | VIM+KPC | VIM+KPC | VIM+KPC |
| 24-011685-01 | VIM+NDM | VIM+NDM | VIM+NDM |
| 22-017330-01 | VIM+NDM | VIM+NDM | VIM+NDM |
| 21-034985-01 | VIM+OXA48 | VIM+OXA48 | VIM+OXA-48-like |

## Supplementary Table 4. AST data for all isolates

| **IHMANumber** | **AMK** | **ATM** | **AZA** | **COL** | **FDC** | **LVX** | **MEM** | **SXT** | **TGC** |  |  |
| --- | --- | --- | --- | --- | --- | --- | --- | --- | --- | --- | --- |
| 24-138113-01 | 8 | >32 | 0.12 | ≤0.25 | | 2 | 2 | 4 | >32 | | 0.5 |
| 24-138114-01 | 4 | 0.12 | 0.06 | ≤0.25 | | 1 | >4 | 2 | >32 | | 1 |
| 24-138116-01 | 2 | 0.12 | 0.06 | ≤0.25 | | 0.12 | 1 | 4 | ≤0.25 | | 0.12 |
| 24-138115-01 | >16 | >32 | 1 | ≤0.25 | | 4 | >4 | 4 | >32 | | 1 |
| 24-138118-01 | 4 | 0.5 | 0.12 | 0.5 | | 1 | >4 | 1 | >32 | | 1 |
| 24-138119-01 | 4 | 32 | 0.06 | ≤0.25 | | 2 | 0.25 | 2 | 0.5 | | 0.5 |
| 24-138120-01 | 16 | >32 | 0.12 | ≤0.25 | | 2 | 2 | 2 | >32 | | 0.5 |
| 24-138121-01 | 2 | 0.06 | 0.03 | ≤0.25 | | 4 | 2 | 4 | 1 | | 0.5 |
| 24-138124-01 | 8 | >32 | 1 | ≤0.25 | | 0.5 | 1 | 1 | >32 | | 0.5 |
| 24-138125-01 | 1 | 1 | 0.5 | ≤0.25 | | 2 | >4 | 0.5 | 0.5 | | 1 |
| 24-011457-01 | 8 | >32 | 0.5 | ≤0.25 | | 1 | 1 | 16 | >32 | | 0.5 |
| 24-011461-01 | 1 | >32 | 0.25 | ≤0.25 | | 1 | >4 | 4 | >32 | | 1 |
| 24-011463-01 | 8 | 0.25 | 0.25 | 0.5 | | 0.5 | 2 | 8 | >32 | | 1 |
| 24-138126-01 | 1 | >32 | 0.25 | ≤0.25 | | 4 | 0.03 | 2 | >32 | | 0.5 |
| 24-138127-01 | 1 | 2 | 0.5 | ≤0.25 | | 1 | >4 | 0.5 | >32 | | 2 |
| 24-138129-01 | >16 | 16 | 0.03 | ≤0.25 | | 0.12 | 0.12 | 0.5 | >32 | | 0.5 |
| 24-011726-01 | 8 | >32 | 0.25 | ≤0.25 | | 4 | 4 | 2 | >32 | | 2 |
| 24-138185-01 | 1 | 0.25 | 0.25 | ≤0.25 | | 1 | 1 | 16 | 1 | | 0.25 |
| 24-138177-01 | 2 | 1 | 0.25 | ≤0.25 | | 1 | >4 | 0.5 | >32 | | 1 |
| 24-138181-01 | 4 | 0.25 | 0.25 | ≤0.25 | | 0.25 | >4 | 8 | 2 | | 4 |
| 24-138182-01 | 1 | >32 | 0.25 | ≤0.25 | | 16 | 0.12 | 2 | >32 | | 4 |
| 24-011657-01 | 8 | 0.12 | 0.06 | ≤0.25 | | 2 | 1 | 16 | >32 | | 0.5 |
| 24-011661-01 | 4 | 0.06 | 0.06 | >8 | | 1 | 1 | 0.5 | 32 | | 0.5 |
| 24-011656-01 | >16 | 0.25 | 0.06 | 0.5 | | 0.5 | 1 | 4 | >32 | | 0.5 |
| 24-011659-01 | 1 | >32 | 0.5 | ≤0.25 | | 8 | 0.12 | 0.5 | >32 | | 0.25 |
| 24-011660-01 | 1 | 32 | 0.5 | 0.5 | | 1 | 1 | 0.25 | 0.5 | | 0.5 |
| 24-011665-01 | 4 | >32 | 1 | ≤0.25 | | 1 | 1 | 2 | >32 | | 0.5 |
| 21-036597-01 | 8 | >32 | 0.5 | ≤0.25 | | 4 | >4 | 1 | >32 | | 1 |
| 21-036655-01 | 1 | >32 | 0.06 | ≤0.25 | | 0.12 | 1 | 0.25 | >32 | | 0.25 |
| 21-036681-01 | 8 | >32 | 0.25 | ≤0.25 | | 2 | >4 | 2 | >32 | | 2 |
| 22-026928-01 | 4 | 2 | 0.12 | ≤0.25 | | 0.5 | 1 | 2 | >32 | | 0.5 |
| 22-033140-01 | >16 | >32 | 0.25 | ≤0.25 | | 4 | 2 | 0.5 | >32 | | 1 |
| 22-033210-01 | 1 | 2 | 0.25 | ≤0.25 | | 0.25 | 0.12 | 4 | 0.5 | | 0.25 |
| 22-063987-01 | 1 | 1 | 0.25 | ≤0.25 | | 0.5 | >4 | 0.5 | >32 | | 2 |
| 22-063999-01 | 2 | 1 | 0.25 | ≤0.25 | | 1 | 4 | 0.5 | >32 | | 2 |
| 24-138108-01 | 2 | >32 | 0.25 | ≤0.25 | | 0.25 | 0.12 | 8 | ≤0.25 | | 0.5 |
| 24-138109-01 | 2 | 0.5 | 0.25 | ≤0.25 | | 1 | 1 | 16 | >32 | | 0.5 |
| 24-138110-01 | >16 | >32 | 0.06 | 0.5 | | 0.25 | 0.06 | 16 | >32 | | 0.5 |
| 24-138111-01 | 1 | 0.5 | 0.03 | ≤0.25 | | 2 | 1 | 8 | ≤0.25 | | 0.5 |
| 24-138107-01 | >16 | >32 | 0.25 | ≤0.25 | | 4 | 4 | 2 | >32 | | 0.25 |
| 24-138186-01 | 4 | >32 | 0.25 | ≤0.25 | | 0.5 | 0.12 | 16 | 0.5 | | 0.25 |
| 24-011662-01 | 4 | 0.5 | 0.12 | ≤0.25 | | 4 | 2 | 64 | >32 | | 2 |
| 24-138190-01 | 1 | >32 | 0.12 | ≤0.25 | | 2 | 2 | 8 | ≤0.25 | | 0.25 |
| 24-138191-01 | 16 | >32 | 0.12 | >8 | | 4 | >4 | 8 | >32 | | 0.5 |
| 24-138192-01 | 8 | >32 | 0.25 | ≤0.25 | | 1 | 4 | 2 | >32 | | 0.5 |
| 21-006683-01 | 2 | >32 | 0.06 | ≤0.25 | | 0.25 | 0.25 | 8 | >32 | | 0.25 |
| 21-016458-01 | >16 | >32 | 2 | 4 | | 0.5 | >4 | >64 | ≤0.25 | | 0.5 |
| 21-016469-01 | >16 | >32 | 0.5 | ≤0.25 | | 4 | >4 | 8 | ≤0.25 | | 0.12 |
| 21-034147-01 | 0.5 | >32 | 0.5 | ≤0.25 | | 2 | >4 | >64 | >32 | | 1 |
| 21-034271-01 | 4 | >32 | 0.25 | ≤0.25 | | 1 | >4 | 8 | >32 | | 1 |
| 21-044328-01 | >16 | >32 | 0.25 | >8 | | 4 | >4 | 64 | 0.5 | | 0.5 |
| 21-044945-01 | 2 | >32 | 2 | ≤0.25 | | 1 | >4 | 16 | >32 | | 1 |
| 22-019431-01 | 1 | >32 | 0.25 | ≤0.25 | | 0.25 | 0.12 | 8 | >32 | | 0.25 |
| 22-020195-01 | 2 | >32 | 0.12 | ≤0.25 | | 1 | >4 | 4 | ≤0.25 | | 0.12 |
| 22-027497-01 | 4 | 32 | 0.06 | ≤0.25 | | 0.5 | >4 | 2 | ≤0.25 | | 0.12 |
| 22-027543-01 | 2 | 32 | 0.06 | ≤0.25 | | 0.5 | >4 | 2 | ≤0.25 | | 0.12 |
| 22-031709-01 | 2 | >32 | 0.5 | ≤0.25 | | 0.5 | >4 | >64 | 1 | | 1 |
| 22-032412-01 | >16 | >32 | 0.06 | ≤0.25 | | 0.5 | >4 | 4 | >32 | | 0.12 |
| 22-042814-01 | 0.5 | >32 | 0.25 | ≤0.25 | | 32 | >4 | 2 | 0.5 | | 1 |
| 22-045588-01 | 4 | 32 | 0.12 | ≤0.25 | | 0.5 | >4 | 8 | >32 | | 0.12 |
| 22-049914-01 | 8 | >32 | 0.12 | ≤0.25 | | 1 | 0.06 | 8 | ≤0.25 | | 0.5 |
| 22-059434-01 | 8 | >32 | 0.25 | >8 | | 4 | >4 | 32 | >32 | | 1 |
| 22-059511-01 | >16 | >32 | 0.25 | ≤0.25 | | 1 | 2 | 16 | 0.5 | | 0.5 |
| 22-059723-01 | 4 | >32 | 0.25 | ≤0.25 | | 0.5 | >4 | 8 | >32 | | 0.5 |
| 22-060022-01 | 1 | >32 | 0.25 | ≤0.25 | | 0.06 | 1 | 4 | >32 | | 0.25 |
| 22-060072-01 | >16 | >32 | 0.25 | >8 | | 4 | >4 | 4 | >32 | | 1 |
| 22-064336-01 | 4 | >32 | 0.12 | ≤0.25 | | 1 | 1 | 1 | >32 | | 0.25 |
| 22-064638-01 | 8 | >32 | 0.25 | >8 | | 0.5 | >4 | 32 | >32 | | 1 |
| 22-064738-01 | >16 | >32 | 0.25 | 2 | | 2 | >4 | 64 | >32 | | 0.5 |
| 22-065838-01 | 1 | >32 | 0.06 | ≤0.25 | | 2 | 0.03 | 4 | >32 | | 0.25 |
| 24-142380-01 | 8 | >32 | 0.5 | ≤0.25 | | 4 | 2 | >64 | 4 | | 1 |
| 22-015013-01 | >16 | >32 | 16 | ≤0.25 | | >64 | 4 | 4 | ≤0.25 | | 0.5 |
| 24-011571-01 | >16 | 8 | 0.12 | ≤0.25 | | 2 | 0.06 | 16 | ≤0.25 | | 0.5 |
| 24-011572-01 | 2 | >32 | 2 | ≤0.25 | | 4 | >4 | 64 | >32 | | 0.5 |
| 24-011575-01 | 16 | 16 | 0.12 | ≤0.25 | | 4 | 0.25 | 8 | 0.5 | | 0.12 |
| 24-011610-01 | 1 | >32 | 1 | ≤0.25 | | 32 | >4 | >64 | >32 | | 0.5 |
| 24-112255-01 | 4 | >32 | 2 | ≤0.25 | | 4 | >4 | 16 | >32 | | 0.25 |
| 21-000953-01 | >16 | >32 | 2 | ≤0.25 | | 2 | >4 | 64 | >32 | | 0.25 |
| 21-000961-01 | 4 | >32 | 8 | ≤0.25 | | 4 | >4 | 32 | >32 | | 0.25 |
| 21-001472-01 | 8 | >32 | 1 | 0.5 | | >64 | >4 | 32 | >32 | | 0.25 |
| 21-001634-01 | >16 | >32 | 0.06 | ≤0.25 | | 1 | >4 | 16 | >32 | | 0.25 |
| 21-002852-01 | 2 | >32 | 0.12 | ≤0.25 | | 1 | >4 | 32 | >32 | | 0.12 |
| 21-003656-01 | >16 | >32 | 2 | 0.5 | | 8 | >4 | 32 | >32 | | 0.25 |
| 21-005535-01 | >16 | 32 | 0.03 | >8 | | 4 | >4 | 8 | 0.5 | | 0.5 |
| 21-007284-01 | 4 | >32 | 1 | ≤0.25 | | 2 | 1 | 16 | >32 | | 0.25 |
| 21-007363-01 | 2 | 16 | 2 | 0.5 | | >64 | >4 | >64 | >32 | | 0.25 |
| 21-013851-01 | 16 | >32 | 1 | ≤0.25 | | >64 | >4 | 64 | >32 | | 0.5 |
| 21-014675-01 | 2 | >32 | 2 | ≤0.25 | | 2 | >4 | 32 | >32 | | 0.25 |
| 21-015422-01 | 8 | >32 | 0.03 | ≤0.25 | | 2 | >4 | 8 | >32 | | 1 |
| 21-015517-01 | 16 | >32 | 1 | ≤0.25 | | 8 | >4 | 64 | >32 | | 0.25 |
| 21-016544-01 | 1 | 4 | 0.25 | ≤0.25 | | 4 | 2 | 16 | ≤0.25 | | 0.25 |
| 21-017194-01 | 4 | >32 | 1 | ≤0.25 | | 8 | >4 | 16 | >32 | | 0.25 |
| 21-018016-01 | 8 | >32 | 0.06 | ≤0.25 | | 2 | >4 | 16 | >32 | | 0.06 |
| 21-032510-01 | 4 | >32 | 0.12 | >8 | | 16 | >4 | >64 | 8 | | 1 |
| 21-038042-01 | 1 | 32 | 1 | ≤0.25 | | 4 | >4 | 32 | >32 | | 0.5 |
| 21-038111-01 | 16 | >32 | 0.25 | ≤0.25 | | 16 | >4 | 64 | >32 | | 1 |
| 21-042158-01 | 4 | >32 | 0.06 | ≤0.25 | | 16 | 1 | 16 | >32 | | 0.25 |
| 21-051301-01 | 4 | 0.25 | 0.06 | ≤0.25 | | 2 | >4 | 16 | ≤0.25 | | 0.12 |
| 22-003858-01 | 2 | >32 | >32 | ≤0.25 | | 4 | >4 | 64 | >32 | | 0.25 |
| 22-009609-01 | 2 | 0.25 | 0.12 | ≤0.25 | | 1 | 0.12 | 32 | ≤0.25 | | 0.12 |
| 22-009658-01 | 4 | >32 | 0.5 | 0.5 | | 32 | >4 | 64 | >32 | | 2 |
| 22-010103-01 | >16 | >32 | 0.03 | ≤0.25 | | 2 | 0.12 | 8 | >32 | | 0.25 |
| 22-010267-01 | 4 | >32 | 2 | ≤0.25 | | 2 | >4 | 32 | >32 | | 0.25 |
| 22-010377-01 | 1 | >32 | 0.06 | ≤0.25 | | 16 | 0.03 | 8 | >32 | | 0.5 |
| 22-011407-01 | >16 | >32 | 0.25 | ≤0.25 | | 2 | >4 | 16 | >32 | | 0.5 |
| 22-012880-01 | 4 | 16 | 8 | ≤0.25 | | 4 | >4 | 64 | >32 | | 0.25 |
| 22-013194-01 | 4 | >32 | 0.12 | >8 | | 2 | >4 | 32 | >32 | | 1 |
| 22-015701-01 | >16 | >32 | 0.5 | ≤0.25 | | 2 | 1 | 32 | >32 | | 0.5 |
| 22-017050-01 | 1 | 32 | 8 | ≤0.25 | | 8 | >4 | 64 | >32 | | 0.5 |
| 22-018987-01 | >16 | 16 | 0.5 | ≤0.25 | | 0.5 | >4 | 16 | >32 | | 0.25 |
| 22-019113-01 | 4 | 16 | 4 | ≤0.25 | | 8 | >4 | 32 | >32 | | 0.12 |
| 22-019425-01 | 2 | >32 | 0.25 | 0.5 | | 64 | 1 | 16 | >32 | | 0.5 |
| 22-019459-01 | 16 | >32 | 0.5 | ≤0.25 | | 4 | >4 | 32 | >32 | | 0.25 |
| 22-020544-01 | 4 | >32 | 0.25 | >8 | | >64 | >4 | 64 | >32 | | 0.5 |
| 22-020561-01 | 4 | >32 | 0.12 | ≤0.25 | | 2 | >4 | 64 | >32 | | 0.5 |
| 22-022186-01 | >16 | >32 | 0.25 | >8 | | 4 | >4 | 16 | >32 | | 1 |
| 22-022205-01 | 4 | >32 | 1 | ≤0.25 | | 4 | >4 | 16 | >32 | | 1 |
| 22-022441-01 | 1 | >32 | 0.12 | ≤0.25 | | 4 | >4 | 64 | >32 | | 0.5 |
| 22-024108-01 | 4 | >32 | 1 | ≤0.25 | | 4 | >4 | 32 | >32 | | 0.25 |
| 22-025210-01 | 2 | >32 | 0.06 | ≤0.25 | | 4 | 1 | 16 | >32 | | 0.25 |
| 22-027729-01 | 4 | 1 | 0.5 | ≤0.25 | | 2 | >4 | 4 | >32 | | 0.5 |
| 22-027750-01 | 2 | 0.5 | 0.12 | ≤0.25 | | 4 | >4 | 32 | >32 | | 0.25 |
| 22-027855-01 | 2 | 0.5 | 0.06 | ≤0.25 | | 4 | 4 | 4 | >32 | | 0.25 |
| 22-028055-01 | 2 | >32 | 2 | ≤0.25 | | 32 | >4 | 64 | >32 | | 0.25 |
| 22-029754-01 | 2 | >32 | 0.25 | ≤0.25 | | 4 | >4 | 2 | >32 | | 0.5 |
| 22-031312-01 | 2 | >32 | >32 | ≤0.25 | | 8 | >4 | 64 | >32 | | 0.5 |
| 22-031313-01 | 2 | >32 | 8 | ≤0.25 | | >64 | >4 | >64 | >32 | | 0.25 |
| 22-031674-01 | >16 | >32 | 8 | ≤0.25 | | 4 | >4 | 32 | >32 | | 0.25 |
| 22-033430-01 | 1 | 0.06 | 0.03 | ≤0.25 | | 2 | 0.12 | 64 | >32 | | 0.5 |
| 22-037259-01 | 4 | >32 | 1 | ≤0.25 | | 8 | >4 | 4 | >32 | | 0.12 |
| 22-041975-01 | 4 | >32 | 0.25 | ≤0.25 | | 2 | 0.03 | 4 | >32 | | 0.25 |
| 22-059178-01 | 16 | >32 | 0.5 | ≤0.25 | | 2 | >4 | >64 | >32 | | 1 |
| 22-059420-01 | >16 | >32 | 0.5 | >8 | | 4 | >4 | >64 | >32 | | 2 |
| 22-060102-01 | >16 | 0.06 | 0.06 | ≤0.25 | | 1 | 1 | 16 | 0.5 | | 0.5 |
| 22-062893-01 | 4 | >32 | 1 | 0.5 | | 8 | >4 | 16 | >32 | | 0.12 |
| 22-064335-01 | 8 | 0.12 | 0.06 | ≤0.25 | | 2 | 0.5 | 4 | >32 | | 0.5 |
| 22-064340-01 | 16 | >32 | 0.25 | 0.5 | | 4 | >4 | 32 | 32 | | 0.5 |
| 22-064341-01 | 4 | >32 | 0.06 | ≤0.25 | | 1 | 1 | 8 | >32 | | 0.5 |
| 22-065120-01 | >16 | >32 | 0.03 | ≤0.25 | | 2 | 1 | 16 | >32 | | 0.25 |
| 22-065727-01 | >16 | 8 | 0.25 | ≤0.25 | | 8 | >4 | 16 | >32 | | 0.5 |
| 22-065995-01 | 2 | >32 | 0.25 | ≤0.25 | | 2 | >4 | 64 | ≤0.25 | | 0.5 |
| 22-076357-01 | >16 | 16 | 0.12 | ≤0.25 | | 4 | >4 | 32 | >32 | | 0.25 |
| 22-081244-01 | 8 | >32 | 4 | ≤0.25 | | 4 | >4 | 64 | ≤0.25 | | 0.5 |
| 22-084982-01 | >16 | 0.25 | 0.12 | ≤0.25 | | 0.5 | >4 | 16 | >32 | | 2 |
| 22-012887-01 | >16 | >32 | 0.12 | ≤0.25 | | 2 | >4 | 32 | >32 | | 0.25 |
| 22-017002-01 | 8 | >32 | 0.5 | ≤0.25 | | 4 | >4 | 16 | >32 | | 0.5 |
| 22-064345-01 | 4 | 32 | 0.03 | ≤0.25 | | 2 | 0.5 | 4 | >32 | | 0.25 |
| 22-037810-01 | 2 | >32 | 0.12 | ≤0.25 | | 2 | 2 | 64 | 4 | | 0.25 |
| 24-011554-01 | 2 | 0.12 | 0.06 | ≤0.25 | | 1 | 1 | 2 | ≤0.25 | | 0.25 |
| 24-037390-01 | >16 | 1 | 0.25 | ≤0.25 | | 8 | >4 | 32 | 1 | | 1 |
| 21-003645-01 | >16 | >32 | 8 | 0.5 | | 16 | >4 | 64 | >32 | | 0.5 |
| 21-043418-01 | ≤0.25 | >32 | ≤0.015 | ≤0.25 | | 1 | >4 | 4 | 2 | | 0.12 |
| 22-014394-01 | 1 | 4 | 1 | ≤0.25 | | 16 | >4 | 64 | >32 | | 0.5 |
| 22-015195-01 | >16 | >32 | 0.5 | ≤0.25 | | 1 | >4 | >64 | >32 | | 1 |
| 22-029567-01 | >16 | >32 | 0.5 | ≤0.25 | | 2 | >4 | >64 | >32 | | 0.5 |
| 22-031656-01 | >16 | >32 | 1 | ≤0.25 | | 4 | >4 | 32 | >32 | | 2 |
| 22-031680-01 | >16 | >32 | 0.5 | ≤0.25 | | 2 | >4 | >64 | 0.5 | | 0.5 |
| 22-031687-01 | 2 | >32 | 1 | 0.5 | | 8 | >4 | >64 | ≤0.25 | | 0.25 |
| 22-064509-01 | 8 | >32 | 0.25 | 0.5 | | 4 | >4 | >64 | 1 | | 0.5 |
| 22-065843-01 | 1 | 0.5 | 0.25 | ≤0.25 | | 1 | >4 | >64 | >32 | | 0.5 |
| 22-066336-01 | >16 | >32 | 0.25 | ≤0.25 | | >64 | >4 | >64 | >32 | | 0.5 |
| 22-076358-01 | >16 | >32 | 0.5 | ≤0.25 | | 2 | >4 | >64 | >32 | | 1 |
| 24-138193-01 | 1 | >32 | 0.5 | ≤0.25 | | 2 | >4 | 8 | ≤0.25 | | 2 |
| 24-011664-01 | 4 | 32 | 0.5 | ≤0.25 | | 4 | >4 | 0.03 | 4 | | 1 |
| 21-000921-01 | 2 | 32 | 0.03 | ≤0.25 | | 1 | 1 | 0.03 | ≤0.25 | | 0.12 |
| 21-001479-01 | 4 | >32 | 0.03 | ≤0.25 | | 0.5 | 0.5 | 0.03 | ≤0.25 | | 0.12 |
| 21-002944-01 | 1 | 0.06 | 0.03 | ≤0.25 | | 0.5 | 1 | 0.03 | >32 | | 1 |
| 21-014805-01 | 4 | >32 | 0.12 | 0.5 | | 0.5 | >4 | 0.03 | >32 | | 0.5 |
| 21-015358-01 | 1 | 0.06 | 0.06 | >8 | | 1 | 0.5 | 0.03 | ≤0.25 | | 0.5 |
| 21-033019-01 | >16 | 8 | 0.25 | ≤0.25 | | 0.12 | >4 | 0.03 | >32 | | 0.5 |
| 21-036163-01 | 1 | 0.06 | 0.03 | ≤0.25 | | 0.5 | 0.03 | 0.03 | ≤0.25 | | 0.5 |
| 21-042123-01 | 4 | >32 | 0.03 | ≤0.25 | | 1 | 0.5 | 0.03 | >32 | | 0.5 |
| 21-043438-01 | 1 | 32 | 0.06 | 0.5 | | 0.5 | >4 | 0.03 | ≤0.25 | | 2 |
| 21-043708-01 | 8 | 16 | 0.03 | ≤0.25 | | 1 | 0.06 | 0.03 | >32 | | 0.12 |
| 22-001213-01 | 1 | >32 | 2 | ≤0.25 | | 2 | 0.25 | 1 | 0.5 | | 1 |
| 22-007797-01 | 4 | >32 | 0.12 | ≤0.25 | | 1 | 4 | 0.03 | >32 | | 2 |
| 22-008965-01 | 4 | >32 | 0.03 | ≤0.25 | | 0.5 | >4 | 0.03 | >32 | | 1 |
| 22-011754-01 | 4 | >32 | 0.03 | ≤0.25 | | 1 | 1 | 0.03 | >32 | | 0.5 |
| 22-015080-01 | 2 | >32 | 0.06 | ≤0.25 | | 0.25 | 1 | 0.03 | >32 | | 0.25 |
| 22-017792-01 | 4 | 0.06 | 0.03 | 0.5 | | 0.06 | 0.06 | 0.03 | ≤0.25 | | 0.12 |
| 22-018989-01 | 0.5 | >32 | 2 | 4 | | 2 | 0.25 | 2 | 0.5 | | 0.5 |
| 22-019446-01 | 2 | 8 | ≤0.015 | ≤0.25 | | 0.25 | >4 | 0.015 | >32 | | 0.06 |
| 22-025265-01 | 4 | 0.12 | 0.12 | >8 | | 0.5 | 0.25 | 0.06 | 0.5 | | 1 |
| 22-029637-01 | 2 | 1 | 0.03 | ≤0.25 | | 0.25 | 0.25 | 0.03 | >32 | | 0.25 |
| 22-042212-01 | 1 | >32 | 0.12 | 0.5 | | 0.5 | 1 | 0.03 | >32 | | 0.25 |
| 22-042705-01 | 4 | >32 | 0.06 | ≤0.25 | | 1 | >4 | 0.03 | >32 | | 0.12 |
| 22-042839-01 | 0.5 | >32 | 4 | ≤0.25 | | 2 | 0.12 | 2 | ≤0.25 | | 0.5 |
| 22-043204-01 | 4 | 32 | 0.12 | ≤0.25 | | 0.5 | >4 | 0.03 | ≤0.25 | | 0.12 |
| 22-062037-01 | 4 | 32 | 0.06 | ≤0.25 | | 0.5 | 1 | 0.03 | >32 | | 0.5 |
| 22-063739-01 | 4 | >32 | 1 | ≤0.25 | | 2 | >4 | 0.5 | >32 | | 1 |
| 22-064031-01 | 8 | >32 | 0.03 | ≤0.25 | | 1 | >4 | 0.03 | ≤0.25 | | 0.12 |
| 22-064053-01 | 2 | >32 | 0.25 | ≤0.25 | | 4 | 4 | 0.03 | >32 | | 0.12 |
| 22-064399-01 | 1 | >32 | 2 | ≤0.25 | | 2 | 0.12 | 2 | ≤0.25 | | 0.5 |
| 22-065184-01 | 4 | >32 | 0.25 | ≤0.25 | | 2 | 4 | 4 | >32 | | 1 |
| 22-065401-01 | 2 | >32 | 1 | ≤0.25 | | >64 | 0.12 | 0.12 | ≤0.25 | | 1 |
| 24-138189-01 | 16 | 8 | 0.5 | ≤0.25 | | 0.5 | >4 | 1 | >32 | | 1 |
| 21-002807-01 | 1 | >32 | 0.12 | ≤0.25 | | 0.5 | >4 | 4 | >32 | | 1 |
| 21-002808-01 | >16 | >32 | 8 | >8 | | 2 | >4 | 32 | >32 | | 0.12 |
| 21-002851-01 | 4 | 8 | 0.25 | ≤0.25 | | 0.25 | >4 | 32 | >32 | | 0.25 |
| 21-015519-01 | 2 | 32 | 0.03 | ≤0.25 | | 1 | >4 | 0.12 | >32 | | 0.25 |
| 21-023317-01 | 1 | 16 | 0.25 | 0.5 | | 0.5 | >4 | 1 | >32 | | 4 |
| 21-036172-01 | 2 | >32 | 2 | ≤0.25 | | 2 | >4 | 2 | >32 | | 0.5 |
| 21-042480-01 | 16 | >32 | 0.25 | ≤0.25 | | 2 | 0.03 | 1 | ≤0.25 | | 0.12 |
| 21-042491-01 | 8 | >32 | 0.06 | ≤0.25 | | 1 | >4 | 0.06 | >32 | | 0.12 |
| 21-043778-01 | 0.5 | 0.25 | 0.25 | 0.5 | | 0.06 | >4 | 16 | 4 | | 0.25 |
| 22-002660-01 | 1 | >32 | 2 | 0.5 | | 2 | 1 | 2 | >32 | | 0.5 |
| 22-003994-01 | 4 | >32 | 16 | ≤0.25 | | 1 | 2 | 0.12 | ≤0.25 | | 0.25 |
| 22-005469-01 | 4 | >32 | 0.25 | ≤0.25 | | 0.5 | >4 | 16 | >32 | | 1 |
| 22-021597-01 | 4 | >32 | 8 | ≤0.25 | | 1 | >4 | 32 | >32 | | 0.25 |
| 22-031320-01 | >16 | >32 | 0.5 | ≤0.25 | | 2 | >4 | 32 | >32 | | 0.5 |
| 22-031470-01 | >16 | >32 | 0.25 | ≤0.25 | | 1 | >4 | 16 | >32 | | 1 |
| 22-032369-01 | 8 | >32 | 1 | ≤0.25 | | 2 | >4 | 2 | >32 | | 2 |
| 22-045348-01 | 4 | >32 | 0.25 | ≤0.25 | | 2 | >4 | 0.25 | ≤0.25 | | 0.12 |
| 22-046687-01 | 4 | >32 | 0.5 | ≤0.25 | | 1 | >4 | 32 | 8 | | 1 |
| 22-046695-01 | 8 | >32 | 0.5 | ≤0.25 | | 2 | >4 | 64 | 8 | | 1 |
| 22-047352-01 | 8 | 16 | 0.06 | ≤0.25 | | 0.5 | 4 | 1 | ≤0.25 | | 0.12 |
| 22-047485-01 | 4 | >32 | 0.5 | 0.5 | | 2 | >4 | 32 | 2 | | 0.25 |
| 22-064398-01 | 8 | >32 | 1 | ≤0.25 | | 1 | >4 | 2 | >32 | | 4 |
| 22-070726-01 | 4 | 8 | 0.25 | ≤0.25 | | 1 | >4 | 2 | >32 | | 1 |
| 22-070727-01 | 8 | >32 | 2 | ≤0.25 | | 2 | >4 | 4 | >32 | | 1 |
| 22-081301-01 | >16 | >32 | 0.5 | 1 | | 2 | >4 | 16 | >32 | | 1 |
| 22-083116-01 | 4 | >32 | 2 | ≤0.25 | | 2 | >4 | 2 | ≤0.25 | | 0.5 |
| 24-138112-01 | 8 | >32 | 0.25 | ≤0.25 | | 0.5 | >4 | 2 | ≤0.25 | | 0.25 |
| 24-138117-01 | 16 | 0.06 | 0.03 | ≤0.25 | | 2 | 0.5 | 8 | >32 | | 0.06 |
| 24-011386-01 | 8 | 8 | ≤0.015 | ≤0.25 | | 1 | >4 | 2 | >32 | | 0.12 |
| 24-138128-01 | 4 | 32 | ≤0.015 | ≤0.25 | | 0.5 | 1 | 2 | >32 | | 0.12 |
| 24-138130-01 | 16 | >32 | 0.25 | ≤0.25 | | 1 | >4 | 4 | >32 | | 0.25 |
| 24-138131-01 | 4 | 0.12 | 0.06 | ≤0.25 | | 0.5 | >4 | 2 | >32 | | 0.12 |
| 24-138184-01 | 8 | 0.06 | 0.06 | >8 | | 2 | 0.12 | 2 | 0.5 | | 0.5 |
| 24-138176-01 | 1 | >32 | 0.5 | >8 | | 32 | 2 | 8 | >32 | | 1 |
| 24-138178-01 | 8 | 4 | 0.25 | ≤0.25 | | 2 | 0.5 | 2 | >32 | | 0.5 |
| 24-138179-01 | >16 | 1 | 0.5 | ≤0.25 | | 0.5 | >4 | 16 | >32 | | 2 |
| 24-138180-01 | 4 | 4 | 0.25 | ≤0.25 | | 1 | 0.5 | 1 | >32 | | 0.5 |
| 24-138183-01 | 4 | >32 | 0.03 | 0.5 | | 4 | 1 | 2 | >32 | | 0.5 |
| 24-138187-01 | 4 | 8 | 0.25 | ≤0.25 | | 4 | >4 | 16 | >32 | | 2 |
| 24-011677-01 | 1 | 0.25 | 0.25 | 0.5 | | 1 | 1 | 32 | >32 | | 4 |
| 24-132736-01 | 8 | >32 | 1 | ≤0.25 | | 2 | 0.12 | 1 | >32 | | 0.25 |
| 24-132744-01 | 4 | 0.25 | 0.12 | ≤0.25 | | 1 | >4 | 4 | >32 | | 0.5 |
| 24-138188-01 | 4 | >32 | 0.06 | ≤0.25 | | 4 | >4 | 4 | >32 | | 0.5 |
| 21-013613-01 | 8 | >32 | 0.25 | 0.5 | | 2 | >4 | 4 | >32 | | 0.5 |
| 21-016542-01 | 1 | >32 | 2 | ≤0.25 | | 2 | >4 | 8 | >32 | | 2 |
| 21-023271-01 | 8 | >32 | 1 | ≤0.25 | | 4 | >4 | 16 | >32 | | 1 |
| 21-038931-01 | 8 | 8 | 0.25 | ≤0.25 | | 2 | >4 | 8 | >32 | | 4 |
| 22-014765-01 | 1 | >32 | 0.12 | 0.5 | | 4 | 0.5 | 4 | >32 | | 0.25 |
| 22-014775-01 | 1 | >32 | 0.06 | ≤0.25 | | 4 | 0.5 | 4 | >32 | | 0.25 |
| 22-015189-01 | 4 | >32 | 0.12 | 0.5 | | 1 | >4 | 1 | >32 | | 0.5 |
| 22-033395-01 | 8 | 8 | 0.25 | ≤0.25 | | 1 | 1 | 32 | >32 | | 0.5 |
| 22-044892-01 | 1 | 32 | 0.25 | ≤0.25 | | 1 | 4 | 2 | >32 | | 0.5 |
| 22-044896-01 | 1 | 16 | 0.5 | ≤0.25 | | 2 | 4 | 2 | >32 | | 0.5 |
| 22-045603-01 | 0.5 | 0.25 | 0.25 | 0.5 | | 1 | 1 | 8 | >32 | | 2 |
| 22-045689-01 | 16 | >32 | 2 | 0.5 | | 1 | >4 | 4 | >32 | | 1 |
| 22-065198-01 | 8 | 0.12 | 0.06 | ≤0.25 | | 2 | 0.06 | 4 | >32 | | 0.5 |
| 22-067242-01 | 1 | 0.25 | 0.12 | ≤0.25 | | 0.5 | >4 | 2 | >32 | | 2 |
| 22-083325-01 | 8 | >32 | 0.12 | 0.5 | | 2 | >4 | 32 | >32 | | 1 |
| 22-083327-01 | 8 | >32 | 0.12 | 0.5 | | 2 | >4 | 16 | >32 | | 0.5 |
| 22-083328-01 | 8 | >32 | 0.12 | 0.5 | | 2 | >4 | 16 | >32 | | 1 |
| 22-083329-01 | 4 | >32 | 0.12 | 0.5 | | 1 | >4 | 16 | >32 | | 2 |
| 22-083330-01 | 8 | >32 | 0.12 | ≤0.25 | | 1 | >4 | 16 | >32 | | 1 |
| 22-083421-01 | 8 | >32 | 0.12 | ≤0.25 | | 2 | >4 | 32 | >32 | | 1 |
| 22-083424-01 | 8 | >32 | 0.12 | ≤0.25 | | 2 | >4 | 32 | >32 | | 1 |
| 22-083426-01 | 8 | >32 | 0.12 | ≤0.25 | | 2 | >4 | 32 | >32 | | 1 |
| 24-142381-01 | 8 | 0.12 | 0.12 | >8 | | 2 | 0.12 | 8 | 0.5 | | 0.25 |
| 24-011533-01 | >16 | >32 | 0.25 | >8 | | 2 | >4 | >64 | >32 | | 1 |
| 22-013158-01 | >16 | >32 | 0.25 | >8 | | 8 | 4 | 64 | >32 | | 4 |
| 22-013188-01 | >16 | >32 | 0.5 | 4 | | 2 | >4 | >64 | >32 | | 1 |
| 22-065888-01 | 8 | >32 | 0.25 | ≤0.25 | | 1 | >4 | 32 | >32 | | 0.5 |
| 24-011685-01 | 2 | >32 | 0.25 | 0.5 | | 1 | 1 | >64 | >32 | | 1 |
| 22-017330-01 | >16 | >32 | 1 | ≤0.25 | | 4 | >4 | 64 | >32 | | 0.5 |
| 21-034985-01 | 2 | >32 | 1 | ≤0.25 | | 4 | >4 | 16 | >32 | | 4 |
